# Supplementary material for: Spatial and temporal population genetic analysis of Semaprochilodus insignis (Prochilodontidae), an overexploited fish from the Amazon basin
Source: PeerJ. 2023 Jun 20;11:e15503. doi: 10.7717/peerj.15503 (PMC10289084; doi:10.7717/peerj.15503)
Supplement: Figure S1 [file peerj-11-15503-s001.pdf]

L(K)

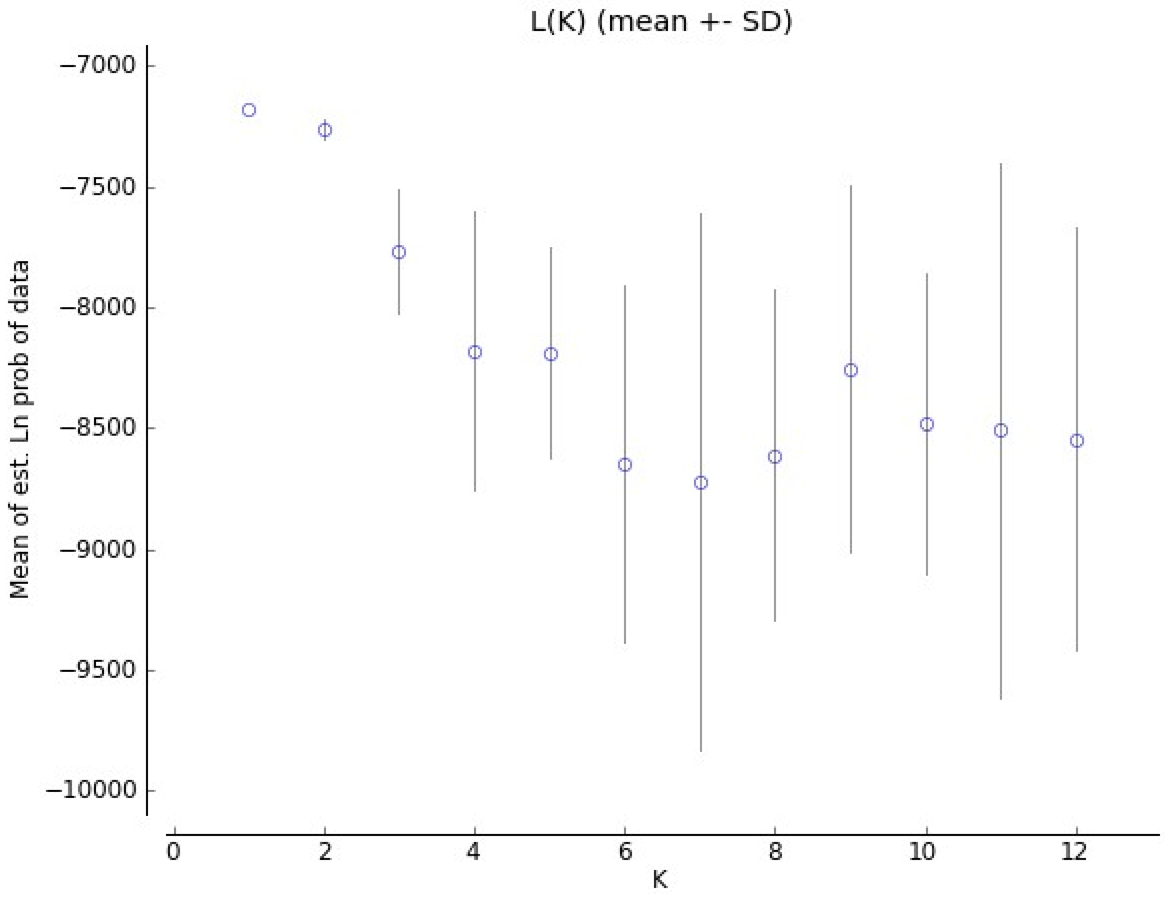

Supplemental Figure S1. The maximum K value estimated for *Semaprochilodus insignis* based in Evanno method.
