# Supplementary material for: Spatial and temporal population genetic analysis of Semaprochilodus insignis (Prochilodontidae), an overexploited fish from the Amazon basin
Source: PeerJ. 2023 Jun 20;11:e15503. doi: 10.7717/peerj.15503 (PMC10289084; doi:10.7717/peerj.15503)
Supplement: Supplementary file 2 [file peerj-11-15503-s002.odt]

Supplemental Table S1. Multiplex systems designed to perform PCR from the combination of the sizes of the alleles.

| Multiplex | Loci | Size range (pb)  described in Passos et al. 2010 | Size range (pb)  observed in this study |
| --- | --- | --- | --- |
| 1 | SinC10 | 99 - 137 | 99 - 139 |
| SinB04 | 159 - 213 | 163 - 207 |
| SinC01 | 355 - 441 | 387 - 445 |
| 2 | SinG08 | 149 - 185 | 152 - 206 |
| SinF05 | 238 - 278 | 232 - 296 |
| 3 | SinC07 | 192 - 220 | 160 - 228 |
| SinE08 | 238 - 279 | 229 - 293 |
|  | SinD03 | 159 - 187 | 146 - 192 |
