# Supplementary material for: Spatial and temporal population genetic analysis of Semaprochilodus insignis (Prochilodontidae), an overexploited fish from the Amazon basin
Source: PeerJ. 2023 Jun 20;11:e15503. doi: 10.7717/peerj.15503 (PMC10289084; doi:10.7717/peerj.15503)
Supplement: Supplementary file 3 [file peerj-11-15503-s003.odt]

Supplemental Table S2. Distribution of the 116 haplotypes of S. insignis, obtained with control region, according to localities. Tab: Tabatinga, Eir: Eirunepé, Tef: Tefé, Coa: Coari, Ppu: Piagaçu-Purus, Mao: Manaus, NAi: Novo Airão, Car: Careiro, San: Santarém, Itai: Itaituba, Jac: Jacareacanga.

| Haplotype | Tab | Eir | Tef | Coa | Pur | Mao | Nai | Car | Sant | Ita | Jac | Total |
| --- | --- | --- | --- | --- | --- | --- | --- | --- | --- | --- | --- | --- |
| H1 | 1 | - | - | - | - | - | - | - | - | - | - | 1 |
| H2 | 1 | 2 | - | - | - | - | - | - | 1 | - | 2 | 6 |
| H3 | 1 | - | - | - | 1 | - | - | - | - | - | - | 2 |
| H4 | 2 | 4 | 2 | 2 | 6 | 4 | 6 | 5 | 5 | 8 | 1 | 45 |
| H5 | 3 | 1 | - | - | 2 | 2 | 3 | - | 1 | - | - | 12 |
| H6 | 1 | 1 | - | - | - | - | - | - | - | - | 1 | 3 |
| H7 | 1 | - | - | - | - | - | - | - | - | - | - | 1 |
| H8 | 1 | - | - | - | - | - | - | - | - | - | - | 1 |
| H9 | 1 | - | - | - | - | - | - | - | - | - | - | 1 |
| H10 | 1 | - | - | - | - | - | - | - | - | - | - | 1 |
| H11 | 1 | - | - | - | - | - | - | - | 1 | 1 | - | 3 |
| H12 | 1 | - | - | - | - | - | - | - | - | - | - | 1 |
| H13 | 2 | - | - | - | - | - | 1 | - | - | - | - | 3 |
| H14 | 1 | - | - | - | - | - | - | - | - | - | - | 1 |
| H15 | - | 1 | 1 | 1 | 1 | 1 | - | 2 | - | 1 | - | 8 |
| H16 | - | 1 | - | - | - | - | - | - | - | - | - | 1 |
| H17 | - | 1 | - | - | - | - | - | - | - | - | - | 1 |
| H18 | - | 1 | - | - | - | - | - | - | - | - | - | 1 |
| H19 | - | 3 | - | - | 1 | - | 1 | - | 1 | - | - | 6 |
| H20 | - | 1 | - | - | 1 | 1 | - | - | - | 2 | 1 | 6 |
| H21 | - | 1 | - | - | 1 | - | - | - | - | - | - | 2 |
| H22 | - | 1 | - | - | - | - | - | - | 1 | - | - | 2 |
| H23 | - | 2 | 1 | - | 1 | - | - | - | 2 | - | - | 6 |
| H24 | - | 1 | - | - | - | - | - | - | - | - | - | 1 |
| H25 | - | 1 | - | - | - | - | - | - | - | - | - | 1 |
| H26 | - | 1 | 2 | - | 2 | 2 | - | 1 | - | - | 3 | 11 |
| H27 | - | 1 | - | - | - | - | - | - | - | - | - | 1 |
| H28 | - | 1 | - | - | - | - | - | - | - | - | - | 1 |
| H29 | - | 1 | - | - | - | - | - | - | - | - | - | 1 |
| H30 | - | 1 | - | - | 1 | - | - | - | 1 | - | 1 | 4 |
| H31 | - | 1 | - | - | - | - | - | - | - | - | - | 1 |
| H32 | - | 1 | - | - | - | - | - | - | - | - | - | 1 |
| H33 | - | 1 | - | - | - | - | - | - | - | 1 | - | 2 |
| H34 | - | 1 | - | - | - | - | - | - | - | - | - | 1 |
| H35 | - | 1 | - | - | - | - | - | 1 | 1 | 1 | - | 4 |
| H36 | - | - | 1 | - | 1 | - | 1 | - | - | - | - | 3 |
| H37 | - | - | 1 | - | - | - | - | - | 1 | - | - | 2 |
| H38 | - | - | 1 | - | - | 1 | 1 | 1 | 1 | - | 1 | 6 |
| H39 | - | - | 1 | - | - | - | - | - | - | - | - | 1 |
| H40 | - | - | 1 | - | - | - | - | - | - | - | - | 1 |
| H41 | - | - | 1 | - | - | - | - | - | - | - | - | 1 |
| H42 | - | - | - | 1 | 1 | - | - | - | - | - | - | 2 |
| H43 | - | - | - | 1 | - | - | - | - | - | - | - | 1 |
| H44 | - | - | - | 1 | 1 | - | - | - | - | - | - | 2 |
| H45 | - | - | - | 1 | - | - | - | - | - | - | - | 1 |
| H46 | - | - | - | 1 | - | - | - | - | - | - | - | 1 |
| H47 | - | - | - | 1 | - | - | - | - | - | - | - | 1 |
| H48 | - | - | - | 1 | - | - | - | - | - | - | - | 1 |
| H49 | - | - | - | - | 1 | - | - | - | - | - | - | 1 |
| H50 | - | - | - | - | 2 | - | 1 | - | 1 | - | - | 4 |
| H51 | - | - | - | - | 2 | - | - | - | - | - | - | 2 |
| H52 | - | - | - | - | 1 | - | - | - | - | - | - | 1 |
| H53 | - | - | - | - | 1 | - | - | - | - | - | - | 1 |
| H54 | - | - | - | - | 1 | - | - | - | - | - | - | 1 |
| H55 | - | - | - | - | 1 | - | - | - | - | - | - | 1 |
| H56 | - | - | - | - | 1 | - | - | - | - | - | - | 1 |
| H57 | - | - | - | - | 1 | - | - | - | - | - | - | 1 |
| H58 | - | - | - | - | 1 | - | - | - | 1 | - | - | 2 |
| H59 | - | - | - | - | 1 | - | - | - | - | - | - | 1 |
| H60 | - | - | - | - | 1 | - | - | - | - | - | - | 1 |
| H61 | - | - | - | - | 1 | - | - | - | - | - | - | 1 |
| H62 | - | - | - | - | 1 | - | - | - | - | - | - | 1 |
| H63 | - | - | - | - | - | 1 | - | - | - | - | - | 1 |
| H64 | - | - | - | - | - | 1 | - | - | - | - | - | 1 |
| H65 | - | - | - | - | - | 1 | - | - | - | - | - | 1 |
| H66 | - | - | - | - | - | 1 | - | - | - | - | - | 1 |
| H67 | - | - | - | - | - | 1 | - | - | - | - | - | 1 |
| H68 | - | - | - | - | - | 1 | - | - | - | - | - | 1 |
| H69 | - | - | - | - | - | 1 | - | - | - | - | - | 1 |
| H70 | - | - | - | - | - | 1 | - | - | - | - | - | 1 |
| H71 | - | - | - | - | - | 1 | - | - | - | - | - | 1 |
| H72 | - | - | - | - | - | 1 | - | - | - | - | - | 1 |
| H73 | - | - | - | - | - | - | 1 | - | - | - | - | 1 |
| H74 | - | - | - | - | - | - | 1 | - | - | - | - | 1 |
| H75 | - | - | - | - | - | - | 1 | - | - | - | - | 1 |
| H76 | - | - | - | - | - | - | 1 | - | - | 1 | - | 2 |
| H77 | - | - | - | - | - | - | 1 | - | - | - | - | 1 |
| H78 | - | - | - | - | - | - | 1 | - | - | - | - | 1 |
| H79 | - | - | - | - | - | - | 1 | - | - | - | - | 1 |
| H80 | - | - | - | - | - | - | 1 | - | - | - | - | 1 |
| H81 | - | - | - | - | - | - | 1 | - | 1 | - | - | 2 |
| H82 | - | - | - | - | - | - | - | 1 | - | - | - | 1 |
| H83 | - | - | - | - | - | - | - | 1 | - | - | - | 1 |
| H84 | - | - | - | - | - | - | - | - | 1 | - | - | 1 |
| H85 | - | - | - | - | - | - | - | - | 1 | - | - | 1 |
| H86 | - | - | - | - | - | - | - | - | 1 | - | - | 1 |
| H87 | - | - | - | - | - | - | - | - | 1 | - | - | 1 |
| H88 | - | - | - | - | - | - | - | - | 1 | - | - | 1 |
| H89 | - | - | - | - | - | - | - | - | 1 | - | - | 1 |
| H90 | - | - | - | - | - | - | - | - | 1 | - | - | 1 |
| H91 | - | - | - | - | - | - | - | - | 1 | - | - | 1 |
| H92 | - | - | - | - | - | - | - | - | 1 | - | - | 1 |
| H93 | - | - | - | - | - | - | - | - | 1 | - | - | 1 |
| H94 | - | - | - | - | - | - | - | - | 1 | - | - | 1 |
| H95 | - | - | - | - | - | - | - | - | 1 | - | - | 1 |
| H96 | - | - | - | - | - | - | - | - | 1 | - | - | 1 |
| H97 | - | - | - | - | - | - | - | - | - | 1 | - | 1 |
| H98 | - | - | - | - | - | - | - | - | - | 1 | - | 1 |
| H99 | - | - | - | - | - | - | - | - | - | 1 | - | 1 |
| H100 | - | - | - | - | - | - | - | - | - | 1 | - | 1 |
| H101 | - | - | - | - | - | - | - | - | - | 1 | - | 1 |
| H102 | - | - | - | - | - | - | - | - | - | 1 | - | 1 |
| H103 | - | - | - | - | - | - | - | - | - | 1 | - | 1 |
| H104 | - | - | - | - | - | - | - | - | - | 1 | - | 1 |
| H105 | - | - | - | - | - | - | - | - | - | 1 | - | 1 |
| H106 | - | - | - | - | - | - | - | - | - | 1 | - | 1 |
| H107 | - | - | - | - | - | - | - | - | - | 1 | - | 1 |
| H108 | - | - | - | - | - | - | - | - | - | 1 | - | 1 |
| H109 | - | - | - | - | - | - | - | - | - | 1 | - | 1 |
| H110 | - | - | - | - | - | - | - | - | - | - | 1 | 1 |
| H111 | - | - | - | - | - | - | - | - | - | - | 1 | 1 |
| H112 | - | - | - | - | - | - | - | - | - | - | 1 | 1 |
| H113 | - | - | - | - | - | - | - | - | - | - | 1 | 1 |
| H114 | - | - | - | - | - | - | - | - | - | - | 1 | 1 |
| H115 | - | - | - | - | - | - | - | - | - | - | 1 | 1 |
| H116 | - | - | - | - | - | - | - | - | - | - | 1 | 1 |
| Total | 14 | 25 | 10 | 9 | 27 | 16 | 16 | 7 | 27 | 20 | 14 |  |
