# Supplementary material for: Spatial and temporal population genetic analysis of Semaprochilodus insignis (Prochilodontidae), an overexploited fish from the Amazon basin
Source: PeerJ. 2023 Jun 20;11:e15503. doi: 10.7717/peerj.15503 (PMC10289084; doi:10.7717/peerj.15503)
Supplement: Supplementary file 4 [file peerj-11-15503-s004.odt]

Supplemental Table S3. Genetic diversity indexes resulting of the 8 microsatellite loci, separately, from the groups of individuals sampled by location. Na: Number of alleles, HO: observed heterozygosity, HE: expected heterozygosity, P: significance level for Hardy Weinberg equilibrium. * Values in bold correspond to significant P values after Bonferroni correction (P<0.000625).

| Localities |  | SinC10 | SinB04 | SinC01 | SinG08 | SinF05 | SinC07 | SinE08 | SinD03 |
| --- | --- | --- | --- | --- | --- | --- | --- | --- | --- |
| Tabatinga | Na | 6 | 10 | 14 | 16 | 15 | 12 | 14 | 12 |
| HO | 0.941 | 0.750 | 0.824 | 0.824 | 0.882 | 0.588 | 1 | 1 |
| HE | 0.775 | 0.762 | 0.936 | 0.911 | 0.948 | 0.847 | 0.905 | 0.922 |
| P | <0.1174 | <0.6201 | <0.0362 | <0.0830 | <0.5253 | <0.0419 | <0.9268 | <0.8474 |
| Eirunepé | Na | 7 | 12 | 16 | 17 | 17 | 14 | 20 | 12 |
| HO | 0.667 | 0.850 | 0.889 | 0.947 | 0.889 | 0.875 | 0.900 | 0.765 |
| HE | 0.837 | 0.846 | 0.932 | 0.947 | 0.913 | 0.895 | 0.955 | 0.881 |
| P | <0.0172 | <0.3279 | <0.4764 | <0.5797 | <0.6328 | <0.7301 | <0.1995 | <0.1089 |
| Tefé | Na | 5 | 6 | 13 | 11 | 12 | 10 | 11 | 10 |
| HO | 0.917 | 0.667 | 0.917 | 1 | 0.818 | 0.750 | 1 | 1 |
| HE | 0.721 | 0.819 | 0.928 | 0.918 | 0.892 | 0.920 | 0.892 | 0.848 |
| P | <0.1851 | <0.4260 | <0.4115 | <0.8638 | <0.3064 | <0.0692 | <0.3362 | <0.9843 |
| Coari | Na | 6 | 6 | 7 | 4 | 10 | 8 | 10 | 8 |
| HO | 0.714 | 0.571 | 1 | 0.333 | 0.833 | 1 | 0.857 | 1 |
| HE | 0.835 | 0.604 | 0.909 | 0.867 | 0.955 | 0.890 | 0.945 | 0.924 |
| P | <0.2594 | <0.6684 | <1 | <0.0681 | <0.2933 | <0.8006 | <0.4164 | <1 |
| Piagaçu-Purus | Na | 7 | 8 | 14 | 18 | 13 | 14 | 18 | 10 |
| HO | 1 | 0.7368 | 0.5790 | 0.8889 | 0.6111 | 0.7778 | 1 | 0.9474 |
| HE | 0.733 | 0.819 | 0.902 | 0.956 | 0.898 | 0.833 | 0.939 | 0.856 |
| P | <0.1428 | <0.0524 | <0.0005 | 0.4472 | <0.0001 | <0.0449 | <0.9348 | <0.4855 |
| Manaus | Na | 10 | 8 | 9 | 15 | 15 | 13 | 17 | 11 |
| HO | 0.850 | 0.800 | 0.563 | 0.563 | 0.750 | 0.882 | 0.889 | 0.800 |
| HE | 0.877 | 0.821 | 0.857 | 0.927 | 0.938 | 0.916 | 0.932 | 0.869 |
| P | <0.0125 | <0.6579 | <0.0224 | <0.0001 | <0.0265 | <0.1415 | <0.4448 | <0.4123 |
| Novo Airão | Na | 5 | 11 | 11 | 12 | 16 | 16 | 17 | 15 |
| HO | 0.600 | 0.952 | 0.762 | 0.700 | 0.810 | 0.882 | 0.900 | 0.952 |
| HE | 0.790 | 0.858 | 0.902 | 0.922 | 0.913 | 0.909 | 0.946 | 0.921 |
| P | <0.0280 | <0.9751 | <0.1250 | <0.0037 | <0.2197 | <0.3728 | <0.0455 | <0.0922 |
| Careiro da Várzea | Na | 6 | 7 | 8 | 11 | 11 | 9 | 11 | 9 |
| HO | 0.778 | 0.889 | 0.556 | 0.875 | 0.889 | 0.875 | 0.625 | 1 |
| HE | 0.856 | 0.850 | 0.817 | 0.950 | 0.941 | 0.933 | 0.950 | 0.902 |
| P | <0.0071 | <0.9943 | <0.0272 | <0.4290 | <0.5635 | <0.5620 | <0.0067 | <1.000 |
| Santarém | Na | 7 | 10 | 14 | 15 | 20 | 11 | 16 | 14 |
| HO | 0.950 | 0.550 | 0.650 | 0.688 | 0.889 | 0.750 | 0.789 | 0.800 |
| HE | 0.824 | 0.705 | 0.899 | 0.946 | 0.957 | 0.844 | 0.923 | 0.881 |
| P | <0.0851 | <0.0093 | <0.0024 | <0.0024 | <0.3664 | <0.2290 | <0.0206 | <0.5080 |
| Itaituba | Na | 8 | 8 | 14 | 8 | 13 | 15 | 18 | 12 |
| HO | 0.765 | 0.667 | 0.778 | 0.231 | 0.765 | 0.882 | 0.889 | 0.938 |
| HE | 0.850 | 0.744 | 0.921 | 0.831 | 0.872 | 0.872 | 0.954 | 0.909 |
| P | <0.3027 | <0.4532 | <0.15 | <0.0001 | <0.0666 | <0.2619 | <0.0360 | <0.3654 |
| Jacareacanga | Na | 10 | 10 | 14 | 18 | 19 | 13 | 22 | 14 |
| HO | 0.765 | 0.765 | 0.588 | 0.882 | 0.882 | 0.846 | 0.882 | 0.938 |
| HE | 0.850 | 0.832 | 0.914 | 0.959 | 0.948 | 0.908 | 0.968 | 0.889 |
| P | 0.5187 | 0.6518 | 0.0013 | 0.1204 | 0.4695 | 0.6645 | 0.1891 | 0.3452 |
| Total number of alleles | 12 | 17 | 26 | 26 | 31 | 29 | 31 | 19 |
| Average number of alleles | 7 | 8.73 | 12.18 | 13.18 | 14.64 | 12.27 | 15.82 | 11.56 |
| Total Expected Heterozygosity | 0.833 | 0.805 | 0.916 | 0.936 | 0.929 | 0.881 | 0.941 | 0.888 |
| Average Expected Heterozygosity | 0.813 | 0.787 | 0.901 | 0.921 | 0.925 | 0.888 | 0.937 | 0.891 |
