# Supplementary material for: Spatial and temporal population genetic analysis of Semaprochilodus insignis (Prochilodontidae), an overexploited fish from the Amazon basin
Source: PeerJ. 2023 Jun 20;11:e15503. doi: 10.7717/peerj.15503 (PMC10289084; doi:10.7717/peerj.15503)
Supplement: Supplementary file 5 [file peerj-11-15503-s005.odt]

Supplemental Table S4. Evanno table output showed only one biological population for Semaprochilodus insignis.

| K | Reps | Mean LnP(K) | Stdev LnP(K) | Ln'(K) | │Ln''(K)│ | Delta K |
| --- | --- | --- | --- | --- | --- | --- |
| 1 | 10 | -7179.290000 | 0.369534 | — | — | — |
| 2 | 10 | -7259.200000 | 42.484010 | -79.910000 | 428.810000 | 10.093445 |
| 3 | 10 | -7767.920000 | 260.254703 | -508.720000 | 97.180000 | 0.373403 |
| 4 | 10 | -8179.460000 | 575.912523 | -411.540000 | 400.560000 | 0.695522 |
| 5 | 10 | -8190.440000 | 435.525257 | -10.980000 | 446.980000 | 1.026301 |
| 6 | 10 | -8648.400000 | 735.431585 | -457.960000 | 385.330000 | 0.523951 |
| 7 | 10 | -8721.030000 | 1111.769063 | -72.630000 | 182.740000 | 0.164369 |
| 8 | 10 | -8610.920000 | 681.354445 | 110.110000 | 246.510000 | 0.361794 |
| 9 | 10 | -8254.300000 | 758.440019 | 356.620000 | 581.320000 | 0.766468 |
| 10 | 10 | -8479.000000 | 623.881744 | -224.700000 | 193.690000 | 0.310459 |
| 11 | 10 | -8510.010000 | 1105.980963 | -31.010000 | 3.070000 | 0.002776 |
| 12 | 10 | -8544.090000 | 876.955143 | -34.080000 | — | — |
