# Supplementary material for: Spatial and temporal population genetic analysis of Semaprochilodus insignis (Prochilodontidae), an overexploited fish from the Amazon basin
Source: PeerJ. 2023 Jun 20;11:e15503. doi: 10.7717/peerj.15503 (PMC10289084; doi:10.7717/peerj.15503)
Supplement: Supplementary file 6 [file peerj-11-15503-s006.odt]

Supplemental Table S5. Estimation of null allele frequencies (Brookfield 1) across studied loci and populations. Note:  Tab: Tabatinga, Eir: Eirunepé, Tef: Tefé, Coa: Coari, Ppu: Piagaçu-Purus, Mao: Manaus, NAi: Novo Airão, Car: Careiro, San: Santarém, Itai: Itaituba, Jac: Jacareacanga. In bold loci with evidence for a null allele.

|  | Tab | Eir | Tef | PPu | Mao | NAi | Car | San | Itai | Jac |
| --- | --- | --- | --- | --- | --- | --- | --- | --- | --- | --- |
| SinC10 | -0,11 | 0,08 | -0,13 | -0,17 | 0 | 0,1 | 0,02 | -0,08 | 0,03 | 0,03 |
| SinB04 | 0 | -0,01 | 0,07 | 0,03 | 0 | -0,06 | -0,05 | 0,08 | 0,03 | 0,02 |
| SinC01 | 0,04 | 0,01 | -0,01 | 0,1593 | 0,1462 | 0,06 | 0,12 | 0,1206 | 0,06 | 0,1586 |
| SinG08 | 0,03 | -0,01 | -0,07 | 0,02 | 0,177 | 0,1047 | 0,01 | 0,1193 | 0,3158 | 0,03 |
| SinF05 | 0,02 | 0 | 0,02 | 0,14 | 0,08 | 0,04 | 0 | 0,02 | 0,04 | 0,02 |
| SinC07 | 0,1282 | 0 | 0,07 | 0,02 | 0 | 0 | 0 | 0,04 | -0,02 | 0,01 |
| SinE08 | -0,07 | 0,02 | -0,08 | -0,04 | 0,01 | 0,01 | 0,1405 | 0,06 | 0,02 | 0,03 |
| SinD03 | -0,06 | 0,05 | -0,1 | -0,06 | 0,03 | -0,03 | -0,08 | 0,03 | -0,03 | -0,04 |
